# Managing Bladder-Neck Stenosis With “Cystoscopy and Proceed” Method: Our Experience

**DOI:** 10.7759/cureus.92494

**Published:** 2025-09-16

**Authors:** Kush Shah, Arya M Kulkarni, Shifa B Karatela, Shikha Patel, Dhruv Patel, Nitya Pathak, Vikky Ajwani, Trisha Ajwani

**Affiliations:** 1 Medicine, Medical College Baroda and Sir Sayajirao General Hospital, Vadodara, IND; 2 Medicine, Gujarat Medical Education &amp; Research Society Medical College and Hospital, Vadnagar, IND; 3 Internal Medicine, Medical College Baroda and Sir Sayajirao General Hospital, Vadodara, IND; 4 Internal Medicine, Gujarat Medical Education &amp; Research Society Medical College and Hospital, Vadodara, IND; 5 Urology, The Cure Urology Hospital, Vadodara, IND

**Keywords:** bladder neck obstruction, cystoscopy, patient centered outcome, patient satisfaction, trans urethral resection of prostate (turp)

## Abstract

Introduction: Bladder-neck stenosis (BNS) is characterized by obstruction of urine flow due to improper relaxation of the bladder neck. Accurate diagnosis through cystoscopy and treatment via bladder-neck incision (BNI) can restore urinary flow and reduce the risk of related complications. Our experience suggests that cystoscopy for diagnosis is underused due to various patient and physician-related factors.

Objective: The primary objective of this study was to evaluate the effectiveness of the "cystoscopy and proceed" method in managing BNS, measured by improvement in uroflowmetry (Qmax). Secondary objectives included assessing recurrence rates, urinary symptom burden using International Prostate Symptom Score (IPSS), patient-reported quality of life Short Form-36 (SF-36), and procedural complications at 10-18 months of follow-up.

Methods: We conducted a retrospective observational study at a private urology center involving 10 patients with cystoscopically confirmed BNS. Data collection included preoperative and postoperative records and follow-up between 10 and 18 months after surgery to assess long-term outcomes in urinary symptoms and satisfaction using IPSS and SF-36, respectively. Statistical analysis was done using Student’s t-test and Pearson's correlation.

Results: The mean age of participants was 64 years. All 10 patients experienced improvements in uroflowmetry, with an average Qmax increase of 14.33 (738.1%), reflecting a 7.4-fold improvement after BNI. A paired t-test revealed a significant difference between preoperative and postoperative Qmax p < 0.001. At 10-18 months follow-up, six patients had satisfaction scores above 80%, and eight scored 70% or higher.

Conclusion: Cystoscopy and BNI effectively manage BNS, leading to significant improvements in patient outcomes and satisfaction.

## Introduction

Bladder-neck stenosis (BNS), also known as bladder-neck contraction (BNC), is a condition characterized by the inability of the bladder neck to properly relax during urination, leading to an obstruction of urine flow. This condition can present either as a primary disorder, referred to as Marrion’s disease, marked by idiopathic fibrosis at the vesicourethral junction, or it may arise secondarily following medical or surgical interventions.

One common cause of secondary BNS is postoperative complications following a transurethral resection of the prostate (TURP), a gold-standard surgical treatment for benign prostatic hyperplasia (BPH) for many years, boasting a success rate of up to 90% [1].

However, despite its effectiveness, BNS is a complication of this procedure, with reported incidence rates ranging from 0% to 4.9% [2]. Other causes include prolonged catheterization, which can lead to urethral strictures and chronic bladder-neck obstruction.

BNS presents with classical urinary obstructive symptoms like heaviness of lower abdomen and feeling of incomplete bladder emptying, increased frequency and urgency of micturition, hesitancy, and incontinence and nocturia. It has an insidious onset as it disguises itself as any other obstructive pathology making it go undiagnosed for a long time even after multiple diagnostic tests such as ultrasonography for imaging of kidneys and bladder, CT scans to check for renal calculi, urine cultures to test for urinary tract infections (UTIs). Urodynamic studies like uroflowmetry typically demonstrates reduced flow but is not diagnostic.

A more accurate diagnostic approach for BNS, as well as for urethral strictures, is cystoscopy, an endoscopic procedure used to visualize the inside of the urinary bladder. Upon diagnosis, treatment typically involves performing bladder-neck incisions (BNIs) at the 5 and 7 o’clock positions using either a bipolar cautery or a Collin’s knife [3]. This procedure generally restores physiological urine flow and can alleviate symptoms rapidly. As a minimally invasive procedure, BNI can expedite the diagnosis of BNS, potentially leading to better patient outcomes by addressing urine voiding issues promptly and preventing serious complications, such as hydronephrosis or, in rare cases, chronic renal failure. Although recurrent or refractory contractures are uncommon, they can cause significant morbidity and greatly impact a patient’s quality of life. For patients who experience incontinence alongside their stenosis, or those with resistant cases of BNS, further treatment options include bladder-neck reconstruction and the possible implantation of an artificial urinary sphincter (AUS) [4].

This article presents the results of a retrospective case series of 10 confirmed cases of BNS, each suffering from similar complaints of incomplete voiding and incontinence, which were diagnosed with cystoscopy and treated with BNIs. This study also focuses on identifying the recurrence (if any) in any of the patient’s previous symptoms with the help of a long term follow up which was conducted within 10-18 months of their intervention for BNS, along with taking into account, the patient satisfaction level related to the procedure and documents any complications they may have experienced.

## Materials and methods

A retrospective observational study was conducted at a privately owned urology center, focusing on 10 patients diagnosed with BNS via cystoscopy, following ethics committee approval. The study included patients with a confirmed diagnosis of BNS, either due to primary causes or as secondary complications from procedures like TURP and other iatrogenic factors. Exclusion criteria involved patients who were unfit for anesthesia, unmarried men (to avoid potential postoperative complications like retrograde ejaculation that could impact future fertility), and individuals with urinary obstructions unrelated to BNS or urethral strictures.

Data collection was done using preoperative and immediate postoperative records of these 10 confirmed cases including chief complaints of the patients, detailed personal and past history, treatment history, lab investigations, preoperative and postoperative uroflowmetry values, postoperative complications (if any), duration of the patient's stay, and recurrence at long term follow up.

A follow-up was conducted 10-18 months after the procedure to assess long-term outcomes, complications, and patient satisfaction. The Short Form-36 (SF-36) was used to evaluate overall patient satisfaction, while the International Prostate Symptom Score (IPSS) assessed urinary symptoms [5,6].

The IPSS includes seven questions on symptoms such as incomplete emptying, intermittency, weak stream, straining to void, frequency, urgency, and nocturia, with each symptom scored from 0 to 5. The total score ranges from 0 to 35, where higher scores indicate more severe symptoms. Scores are categorized as mild (1-7), moderate (8-19), or severe (20-35) [6].

The SF-36 questionnaire assesses patient health across eight domains, including physical functioning (10 items), bodily pain (two items), role limitations due to physical health problems (four items), role limitations due to personal or emotional problems (four items), emotional well-being (five items), social functioning (two items), energy/fatigue (four items), and general health perceptions (five items), with scores ranging from 0 to 100 for each domain, where higher scores indicate better health, out of which five relevant to our study were used, namely, physical functioning, role limitations due to physical health, emotional well-being, pain, and general health [5].

Quantitative variables like age, BMI, preoperative and postoperative Qmax, and IPSSes were expressed as means. Statistical analysis included t-tests to compare preoperative and postoperative Qmax, and Pearson's correlation to assess the relationship between IPSS, Qmax improvement, and variables like age and BMI. Change in Qmax postoperatively was considered as the primary outcome and relation of patient characteristics with IPSS and SF-36 scoring postoperatively were considered as secondary outcomes.

**Figure 1:**
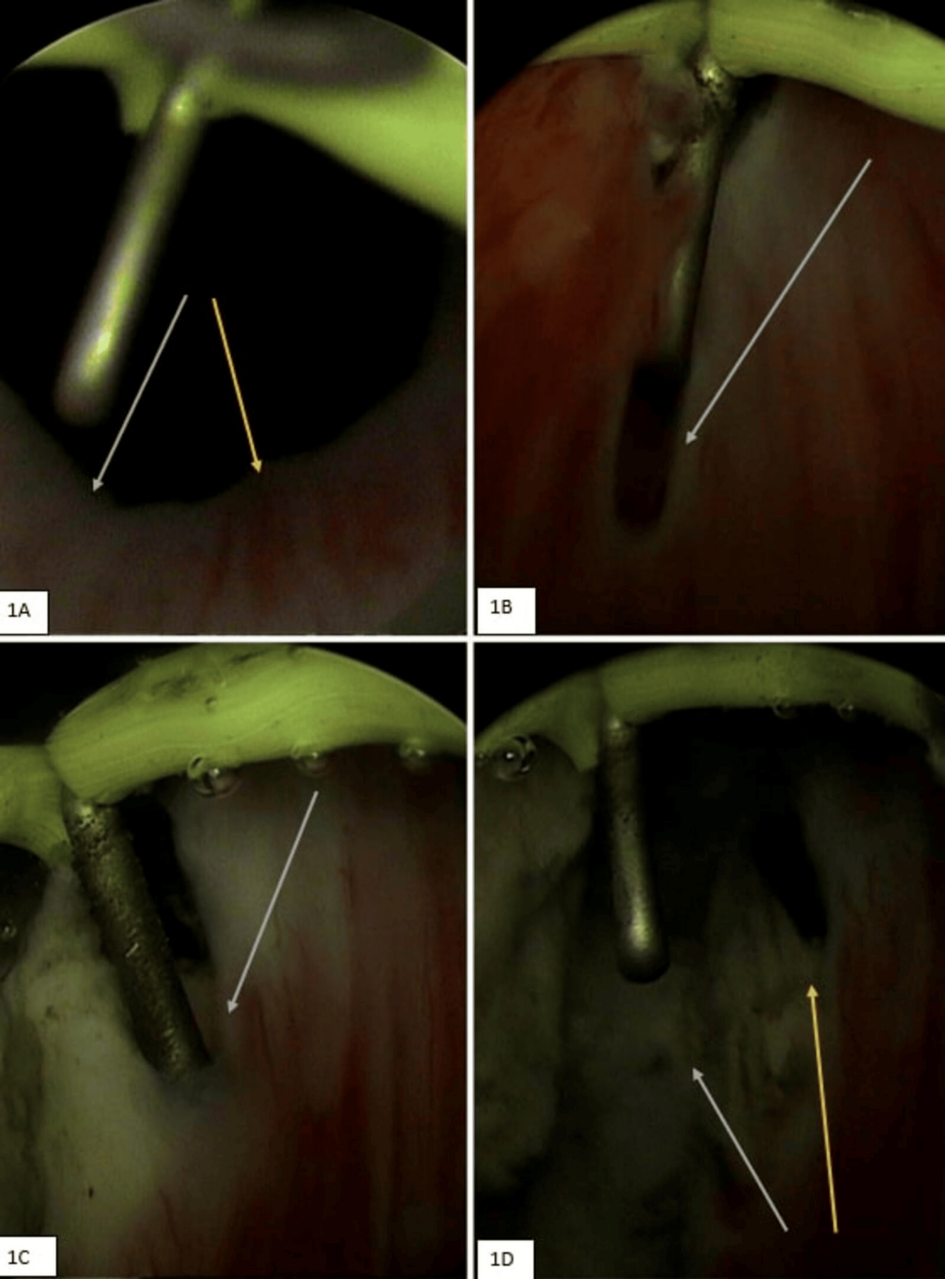
Preoperative, intraoperative, and postoperative views of bladder-neck obstruction and surgical incisions 1A: Preoperative view of bladder neck depicting bladder-neck obstruction (white and yellow arrows). 1B: Intraoperative image demonstrating 7 o’clock incision for BNS (arrow). 1C: Intraoperative image demonstrating 5 o’clock incision for BNS (arrow). 1D: Immediate postoperative image depicting 5 o'clock (yellow arrow) and 7 o’clock (white arrow) incision with relief in bladder-neck obstruction. BNS: Bladder-neck stenosis

Figure 1 illustrates the step-by-step procedure of endoscopic treatment for bladder-neck obstruction. In the initial picture (1A), the bladder neck is highly constricted, obviously indicating the site of obstruction. During surgery, precise incisions are performed-initially at the 7 o'clock position (1B), followed by the 5 o'clock position (1C) to slowly open up the obstructed region. The last image (1D) demonstrates a significantly wider and more distinct bladder neck, indicating that the procedure effectively alleviated the obstruction and restored normal flow (Figure 1).

## Results

Case presentation

Case 1

A 67-year-old male patient presented with a five-month history of voiding difficulty, characterized by a weak stream and burning micturition. Ultrasound kidney, ureter, and bladder (USG KUB) revealed post-void residual volume, and retrograde urethrography showed bladder-neck constriction and urethral stricture. The patient underwent rigid cystoscopy, direct visual internal urethrotomy, BNI at 5 and 7 o'clock using bipolar electrocautery, and urethral dilatation under spinal anesthesia. Postoperative uroflowmetry (Qmax) improved from 5 to 14 ml/s. He was advised to return for follow-up and non-invasive urethral dilatation with a 30 Fr catheter, performed weekly over four-week intervals to prevent recurrence.

Case 2

A 63-year-old male patient presented with voiding difficulty, burning micturition, intermittent lower abdominal pain in the right iliac quadrant, and mild fever. Urinalysis revealed a bacterial UTI, and blood tests showed hyperkalemia with elevated creatinine but preserved estimated glomerular filtration rate (eGFR). Imaging ruled out kidney stones or prostate abnormalities. The patient was treated with intravenous meropenem and calcium polystyrene sulfonate for UTI and hyperkalemia. Preoperative uroflowmetry showed a Qmax of 4.4 ml/s. Rigid cystoscopy revealed bladder-neck obstruction and BNIs were performed using electrocautery. Postoperative Qmax improved to 18.1 ml/s.

Case 3

A 75-year-old male patient presented with a six-month of suprapubic pain and incomplete voiding, and one month of involuntary urine dribbling was diagnosed with bladder-neck obstruction and urethral strictures via USG KUB and ascending urethrography. Preoperative uroflowmetry (Qmax) was 3.5 ml/s. Cystoscopy confirmed BNS, and BNIs were performed under spinal anesthesia. Postoperative Qmax improved to 15.8 ml/s with no recurrence.

Case 4

A 61-year-old male patient presented with a two-month history of weak urine stream and 10 days of severe left lumbar pain, nausea, and vomiting. USG KUB showed post-void residual volume and multiple left ureteric calculi. Retrograde urethrography was inconclusive due to a suspected prostatic urethral obstruction. Laboratory tests were consistent with pyelonephritis, with elevated leukocyte count, neutrophils, erythrocyte sedimentation rate (ESR), and C-reactive protein (CRP). Preoperative uroflow (Qmax) was 1.2 ml/s. The patient underwent ureteroscopy and double J (DJ) stenting, but advancement of the ureteroscope access was restricted by BNS and prostatic urethral strictures. Retrograde manipulation of the calculi released purulent urine, confirming severe pyelonephritis. Therefore, percutaneous nephrolithotomy was deferred until the infection was treated. Bladde-neck incisions were made using bipolar electrocautery. Extracorporeal shock wave lithotripsy was scheduled for the following week. Postoperative uroflow improved to 17.5 ml/s.

Case 5

A 62-year-old male patient presented with urine retention, passing only a few drops for one month, and severe suprapubic pain for 10 days. USG KUB revealed a distended bladder, and ascending urethrography showed bladder-neck constriction. Preoperative uroflow was 1.2 ml/s. Due to his ischemic heart disease, the patient was managed conservatively. Since catheterization was not feasible due to urethral stricture, Urethral stricture and bladder-neck obstruction were treated with direct visible internal urethrotomy, cystoscopy, and BNIs under spinal anesthesia. Postoperative uroflow improved to 16.6 ml/s.

Case 6

A 24-year-old male patient presented with a weak and intermittent urine stream, burning micturition, and urinary retention for three months. USG KUB and prostate sonogram showed bladder distension after voiding, and ascending urethrography revealed significant urethral narrowing and bladder-neck contracture. Preoperative uroflowmetry (Qmax) was 8.3 ml/s. Rigid cystoscopy confirmed vesicourethral stricture and BNS. Treatment included direct visual internal urethrotomy and BNIs at the 5 and 7 o'clock positions using electrocautery via Collin’s knife under general anesthesia. Postoperative uroflowmetry improved to 25.4 ml/s.

Case 7

A 53-year-old male patient presented with difficulty urinating, a weak stream, incomplete voiding, and nocturnal enuresis for one month had a preoperative uroflowmetry (Qmax) of 0.8 ml/s. This suggested lower urinary tract obstruction. Rigid cystoscopy revealed BNS and urethral stricture, which were treated with deep BNIs at the 5 and 7 o'clock positions using a Collin’s knife. Postoperative uroflowmetry improved to 20.9 ml/s. The patient was hospitalized for two days.

Case 8

A 75-year-old male patient presented with hematuria, difficulty urinating, incomplete bladder emptying, and urinary retention, worsening over three weeks. He had a history of TURP for BPH and aortic valve replacement. Clinical investigations, including blood tests and imaging, suggested a recurrent BPH with an echogenic prostate lesion on the prostate sonogram and elevated prostate-specific antigen (PSA) levels.

Preoperative uroflowmetry showed a Qmax of 2.7 ml/s and an IPSS of 18. Cystoscopy revealed BNS and urethral strictures, which were treated with BNIs and urethral dilatation. TURP was performed, revealing and removing a large obstructive median lobe. Postoperative uroflowmetry improved to 17.5 ml/s.

Case 9

A 68-year-old male patient presented with difficulty voiding, reduced urinary flow, and incomplete bladder emptying. He presented after one month of symptoms and 10 days of suprapubic pain. He had a history of prostate cancer treated with radical prostatectomy, radiation, and hormone therapy. USG KUB showed residual urine, and retrograde urethrography was inconclusive due to multiple strictures. Preoperative uroflowmetry showed a Qmax of 4 ml/s. Direct visual internal urethrotomy and rigid cystoscopy diagnosed bladder-neck contracture, treated with endoscopic BNIs at 5 and 7 o'clock positions using bipolar electrocautery. Postoperative uroflowmetry improved to 17 ml/s. The patient was observed for one day and experienced symptom recurrence. A repeat cystoscopy nine months later confirmed recurrent BNS, which was treated with additional BNIs.

Case 10

A 92-year-old male patient presented with urinary retention, incomplete bladder emptying, and burning micturition for the past two months. Preoperative uroflowmetry showed a Qmax of 3.8 ml/s. Rigid cystoscopy confirmed bladder-neck obstruction and urethral strictures. The patient underwent direct visual internal urethrotomy with deep BNIs at the 5 and 7 o'clock positions using electrocautery under spinal anesthesia. Postoperative uroflowmetry improved to 15.4 ml/s.

Primary outcome

**Figure 2:**
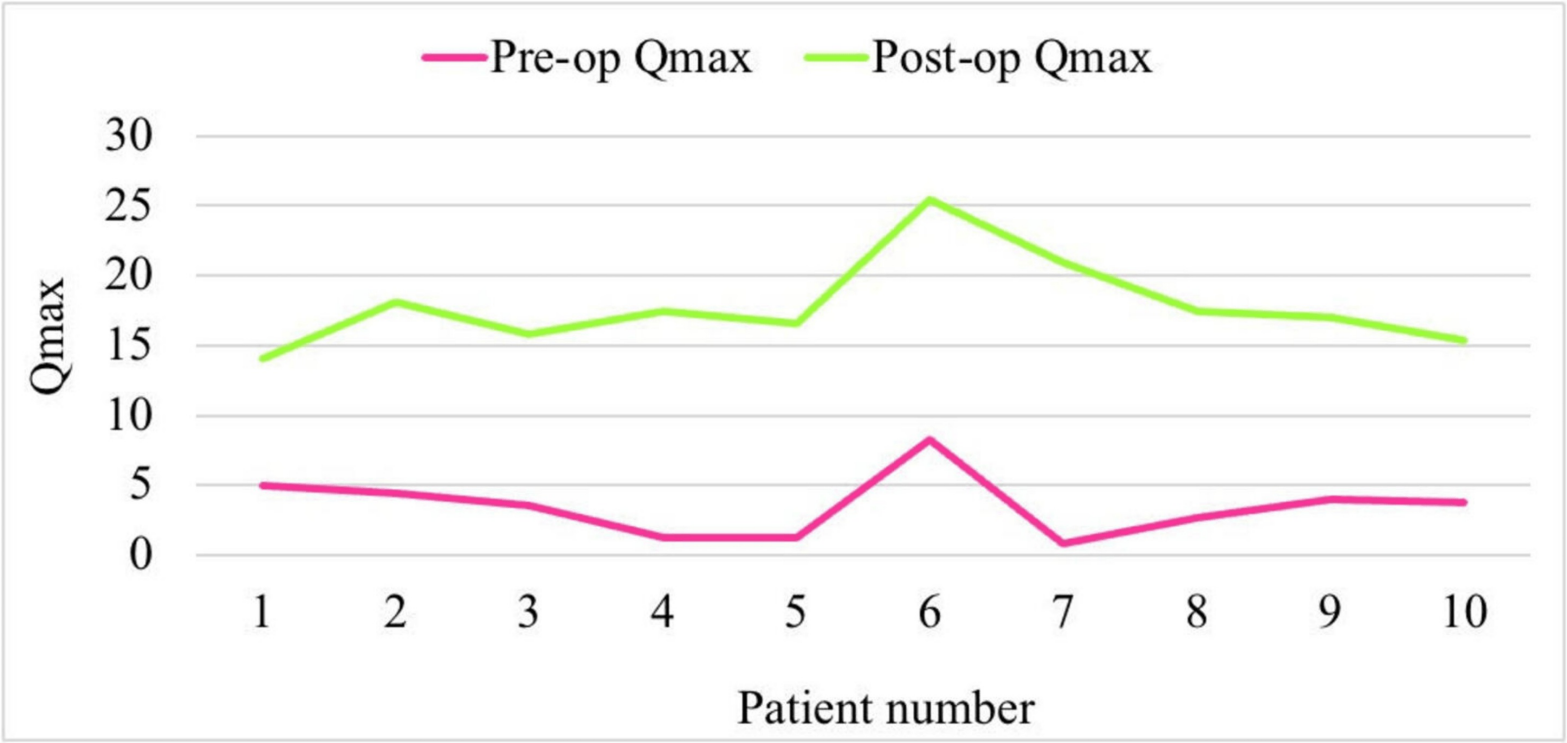
Preoperative and postoperative uroflowmetry finding (Qmax) of 10 patients

The mean age of participants in our study was 64 years. All 10 patients experienced improvements in their uroflowmetry scores, with an average increase in Qmax of 14.33 ml/s (738.1%), reflecting a 7.4-fold improvement following BNI, as detailed in Table 1 and Figure 2. A paired t-test revealed a significant difference between preoperative Qmax (M = 3.5, SD = 2.2) and postoperative Qmax (M = 17.8, SD = 3.2), with t (9) = 14.5 and p < 0.001.

**Table 1:** Clinical and procedure characteristics of patients Qmax: Maximum flow rate; IPSS: International Prostate Symptom Score

Sr. no.	Age (years)	BMI (kg/m^2^)	Preop Qmax (ml/sec)	Postop Qmax (ml/sec)	Increase in Qmax (ml/sec)	% Increase in Qmax	Postop hospital stay (days)	Follow-up (months)	IPSS
1	67	23.5	5	14	9	180.0	2	13	3
2	63	25	4.4	18.1	13.7	311.4	1	18	4
3	75	22	3.5	15.8	12.3	351.4	2	16	2
4	61	21.3	1.2	17.5	16.3	1358.3	2	12	5
5	62	27	1.2	16.6	15.4	1283.3	4	14	2
6	24	19.7	8.3	25.4	17.1	206.0	2	10	1
7	53	23	0.8	20.9	20.1	2512.5	2	15	4
8	75	23	2.7	17.5	14.8	548.1	3	13	3
9	68	20.1	4	17	13	325.0	1	12	10
10	92	21	3.8	15.4	11.6	305.3	5	14	6
Mean	64.0	22.6	3.5	17.8	14.3	738.1	2.4	13.7	4.0
SD	17.6	2.2	2.2	3.2	3.1	756.5	1.3	2.3	2.6

**Figure 3:**
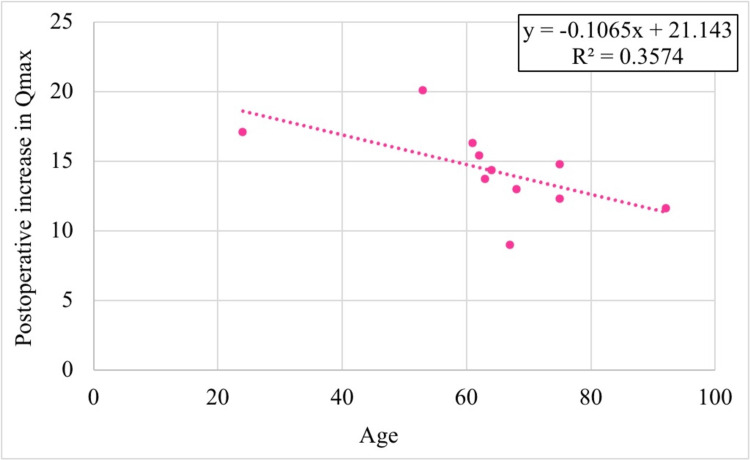
Correlation of age with increase in uroflowmetry finding (Qmax)

**Figure 4:**
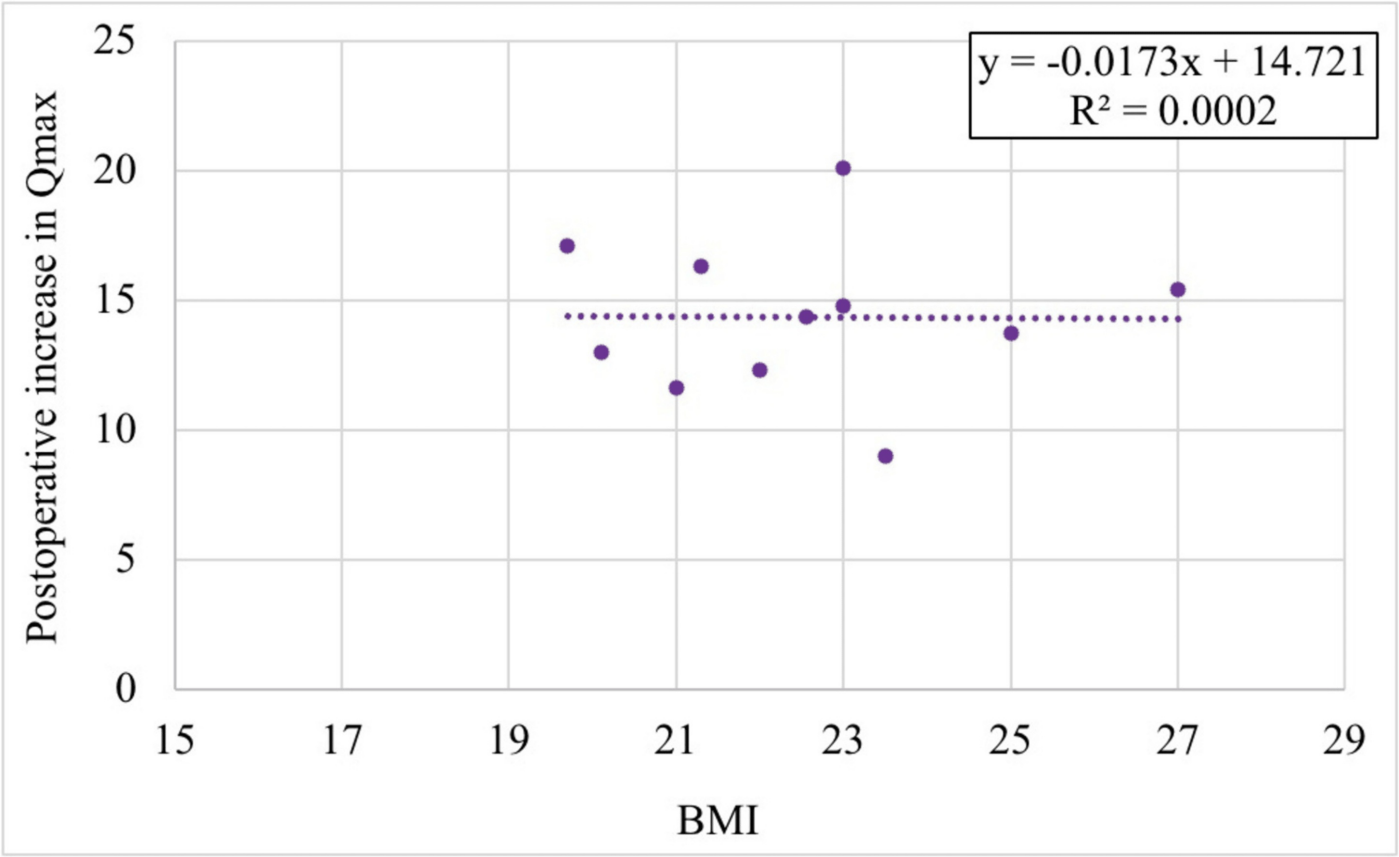
Correlation of BMI with an increase in uroflowmetry finding (Qmax)

Age and BMI were negatively correlated with postoperative Qmax improvement, although these correlations were statistically non-significant (r(8) = -0.598, p = 0.068 for age, and r(8) = -0.0124, p = 0.973 for BMI). As depicted in Figures 3, 4, the R² values indicated that age accounted for 35% of the variance in Qmax improvement, while BMI accounted for only 0.2%.

Secondary outcomes

Relation of Patient Characteristics with IPSS

**Figure 5:**
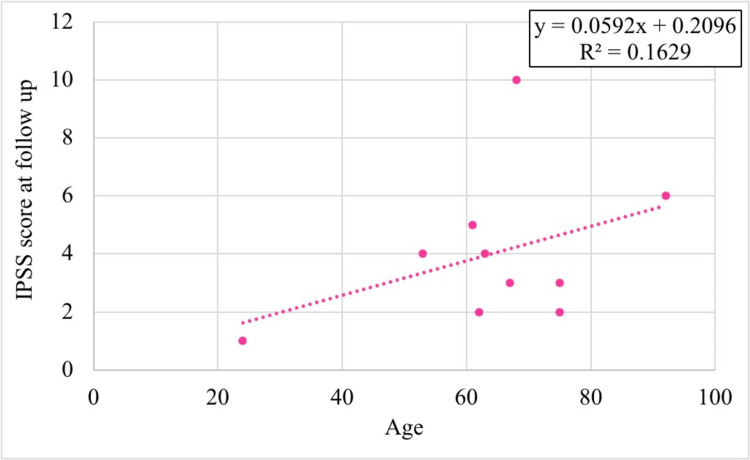
Correlation of age with IPSS at long-term follow-up IPSS: International Prostate Symptom Score

**Figure 6:**
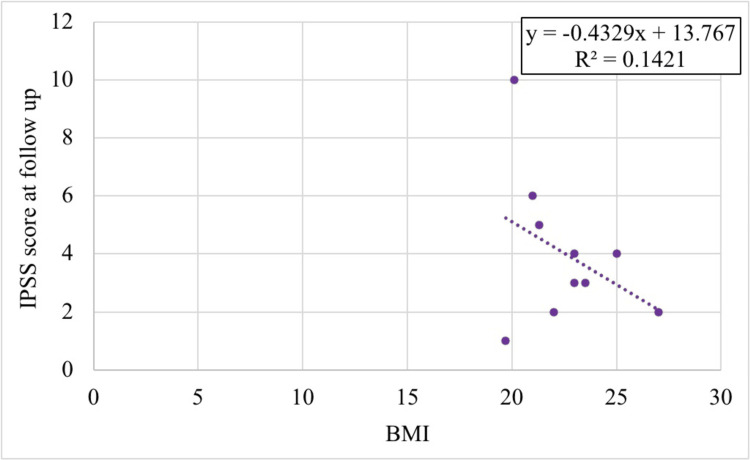
Correlation of BMI with IPSS at long-term follow-up IPSS: International Prostate Symptom Score

Age was positively correlated with the IPSS at long-term follow-up (r(8) = 0.404, p = 0.247), while unexpectedly, BMI showed a negative correlation (r(8) = -0.377, p = 0.283). Both correlations were statistically non-significant, as shown in Figures 5, 6.

Postoperative SF-36 Scoring

**Figure 7:**
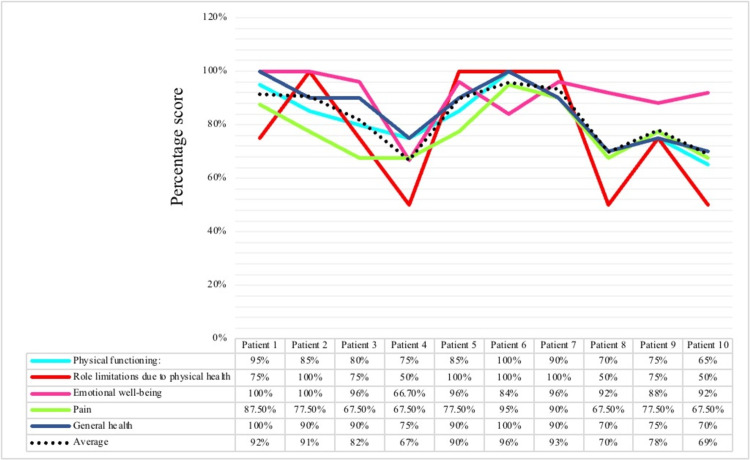
Scoring of patient satisfaction by SF-36 criteria SF-36: Short Form-36

Six out of the 10 patients achieved a cumulative average score above 80%, and eight patients had scores of 70% or higher. The lowest cumulative score was 69%, observed in patient 10, which may be partially attributed to his advanced age of 92 years (Figure 7).

## Discussion

BNS frequently occurs as a postoperative complication, particularly following TURP for BPH and other iatrogenic procedures. The subtle and gradual onset of BNS often leads to delayed diagnosis, which can result in considerable patient discomfort, anxiety, and potentially serious renal complications.

Typically, BNS is diagnosed by low urinary flow rates combined with radiographic evidence of obstruction [7]. However, imaging methods like X-rays and ultrasounds can be misleading or inconclusive in some cases. As such, cystoscopy, along with uroflowmetry, remains the most effective diagnostic tool for BNS [8]. Despite its effectiveness, cystoscopy is often underutilized due to its cost and invasive nature, which may cause patient hesitation. This reluctance can delay diagnosis and treatment. Additionally, some physicians may avoid recommending cystoscopy, which can result in patients presenting with more severe complications that could have been prevented through timely intervention with BNI.

Initial treatment for BNS should involve endourological methods such as dilatation, laser incision, or resection. If three attempts fail, open reconstruction is advised. Various surgical techniques, including abdominal, perineal, and abdominoperineal approaches, have been successful. In cases of persistent BNS, open reconstruction is recommended, with YV-plasty as a common method and T-plasty as a variation. Both procedures have high success rates. Robot-assisted reconstructive options have also been reported for BNS [9,10].

In this study, we report our experience with 10 patients, eight of whom (80%) were discharged within two days postoperatively. Our findings demonstrate that cystoscopy and BNI were performed successfully, resulting in high patient satisfaction, as measured by the SF-36, and significant symptom improvement, as reflected in the IPSS at long-term follow-up of 10-18 months. There was a statistically significant postoperative increase in Qmax, which indicates improved urinary flow, however, it was negatively correlated with age and BMI, suggesting that younger patients with lower BMI experienced better outcomes. However, these correlations were not statistically significant. Similarly, while age showed a positive correlation with IPSSes and BMI a negative one, these relationships also lacked statistical significance.

In conclusion, for the prompt diagnosis and effective management of BNS, adopting a "cystoscopy and proceed" approach should be considered for non-refractory cases. This study highlights the procedure's minimal invasiveness and ease, along with its potential to improve patient prognosis and quality of life. We recommend performing a cystoscopy when BNS is suspected, as it can prevent further complications and streamline treatment for both patients and physicians.

Limitations

Our study had a small sample size, which made it challenging to draw definitive conclusions. Additionally, the underutilization of cystoscopy for diagnosing BNS was based on our personal observations. The follow-up period was limited to 18 months due to the study's scope, which may not capture long-term outcomes. Future research should explore the barriers that patients and physicians face in utilizing cystoscopy for BNS diagnosis and treatment, as addressing these issues could improve early detection and management.

## Conclusions

Cystoscopy and BNI are effective and minimally invasive approaches for treating BNS, leading to significant symptom resolution and high patient satisfaction. Despite the limited sample size, our findings suggest that timely diagnosis and intervention can prevent the progression of complications associated with BNS. We recommend that cystoscopy be more widely considered when BNS is suspected, as it offers a reliable diagnostic tool and a path to successful treatment. Future research should focus on addressing the barriers to the utilization of cystoscopy to further enhance patient outcomes and care quality.
